# Elevated high-sensitive cardiac troponin T in emergency department patients: insights from a retrospective descriptive cohort study

**DOI:** 10.1186/s12245-024-00735-w

**Published:** 2024-10-07

**Authors:** Finn Syryca, Bernhard Haller, Lisa Schmid, Christiane Kallweit, Philipp Nicol, Teresa Trenkwalder, Karl-Georg Kanz, Anja Haas, Michael Dommasch

**Affiliations:** 1 Department of Cardiology, German Heart Center Munich, Technical University of Munich, Munich, Germany; 2 Institute for AI and Informatics in Medicine, Klinikum rechts der Isar, Technical University of Munich, Munich, Germany; 3 Emergency Department, Klinikum rechts der Isar, Technical University of Munich, Ismaninger Straße 22, 81675 Munich, Germany; 4 Department of Cardiology, Klinikum rechts der Isar, Technical University of Munich, Munich, Germany

**Keywords:** Emergency department, High-sensitive troponin, Percutaneous coronary intervention

## Abstract

**Background:**

High-sensitive cardiac troponin T (hs-cTnT) assessments are routinely conducted in German emergency departments (EDs). However, data describing a large number of ED patients with pathological hs-cTnT levels and subsequent clinical outcomes are limited.

**Methods:**

This retrospective descriptive analysis included 141.892 patients who presented to the interdisciplinary ED at Klinikum rechts der Isar in Munich, Germany, between January 2019 and December 2021. Patients with trauma diagnoses were excluded, focusing on those with elevated hs-cTnT levels. These patients were categorized into three groups based on the International Classification of Procedures in Medicine (ICPM): those with elevated hs-cTnT who received no coronary angiography (NCA), those who underwent diagnostic coronary angiography (DCA), and those who received percutaneous coronary intervention (PCI). The objective of this study was to characterize a large emergency department patient cohort and assess their subsequent clinical outcomes.

**Results:**

After initial Manchester Triage Sytem (MTS) categorization, 32.6% (46.307/141.892) of patients were identified as non-trauma cases. Of these, 9.9% (4.587/46.307) had hs-cTnT levels exceeding 14 ng/L. Within this subset, 70.4% (3.230/4.587) did not undergo coronary angiography, 15.4% (705/4.587) underwent DCA and 14.2% (652/4.587) received PCI. Chest pain occurred more frequently in the PCI group (28.0%, 160/652) compared to the DCA group (18.3%, 113/705) or NCA group (5.7%, 159/3230), *p* < 0.001. However, breathing problems occurred more frequently in the NCA group (23.2%, 647/3230) compared to the PCI group (17.7%, 101/652) or DCA group (21.8%, 135/705), *p* < 0.001. Also, collapse was more frequent in patients in the NCA group (4.0%, 112/3230) compared to the DCA group (3.4%, 21/705) or PCI group (3.5%, 20/652), *p* < 0.001. Overall, in-hospital mortality was significantly higher in the NCA group (7.9%, 256/3230) compared to the DCA group (2.3%, 16/705) or PCI group (4.1%, 27/652), *p* < 0.001.

**Conclusion:**

Emergency patients with elevated hs-cTnT who did not undergo coronary angiography faced a higher risk of in-hospital mortality in our retrospective descriptive study. Given the heterogeneous nature of presenting complaints in emergency departments, identifying at-risk patients can pose challenges for treating physicians.

**Supplementary Information:**

The online version contains supplementary material available at 10.1186/s12245-024-00735-w.

## Introduction

In recent years, the number of patients presenting to emergency departments (EDs) has increased significantly, highlighting the challenge of potential overcrowding in these critical care settings [1]. In this context, rapid and effective patient management is essential to expedite therapeutic interventions and ensure optimal patient outcomes. Acute myocardial infarction (AMI) remains one of the leading causes of mortality in developing countries [2]. Chest pain, a hallmark symptom of AMI, is a prevalent reason for ED visits in developed nations [3]. Pathologically elevated levels of high-sensitive troponin (hs-cTnT) are closely associated with an increased risk of AMI [4], and as a result, hs-cTnT measurements are routinely conducted in clinical practice. In interdisciplinary EDs, physicians frequently face the critical decision of whether to pursue ambulatory or inpatient care for patients with elevated hs-cTnT, and whether these patients may require percutaneous coronary intervention (PCI). Despite the routine use of hs-cTnT measurements, the implications and consequences of elevated hs-cTnT levels in the ED setting often remain unclear without a comprehensive understanding of their significance for the patient’s prognosis and clinical outcome. Currently, data evaluating the impact of pathological hs-cTnT levels on subsequent clinical developments are limited. Given this gap in knowledge, we aimed to characterize a large cohort of emergency patients and assess their subsequent clinical outcomes.

## Patients and methods

### Study design and population

**Fig. 1:**
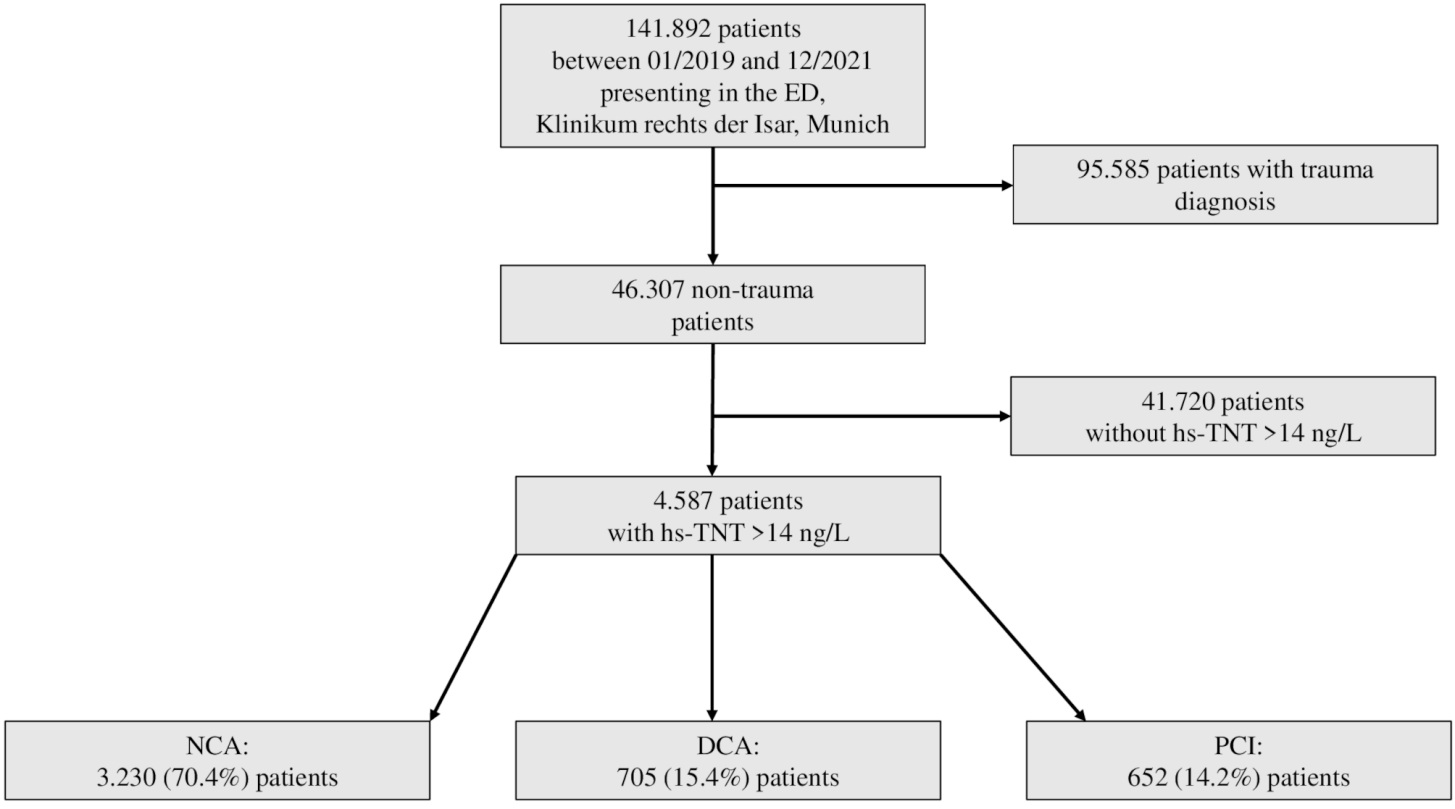
Entire population during the inclusion period and detailed exlusion criteria. Abbreviations: NCA, no coronary angiography; DCA, diagnostic coronary angiography; ED, emergency department; hs-cTnT, high-sensitive troponin; PCI, percutaneous coronary intervention;

This retrospective descriptive study was conducted at the interdisciplinary ED of Klinikum rechts der Isar in Munich, Germany. The study encompassed a total of 141.892 patients who presented between January 2019 and December 2021. To refine the study population, patients diagnosed with trauma (*n* = 95.585) and those without elevated hs-cTnT levels (*n* = 41.720) were excluded. The final cohort consisted of 4.587 patients who had hs-cTnT levels of > 14 ng/L. The patients were categorized based on the procedures they underwent according to the International Classification of Procedures in Medicine (ICPM), with data extracted from our clinical information system, i.s.h.med^®^ (Cerner Corporation, The Hague, Netherlands). The categories were: No coronary angiography (NCA, 3.230 patients), diagnostic coronary angiography (DCA, 705 patients) and percutaneous coronary intervention (PCI, 652 patients). A detailed flowchart illustrating the inclusion and exclusion criteria can be found in Fig. 1. Baseline characteristics of the study population are summarized in Table 1, while presenting complaints are detailed in Table 2.

**Table 1:** Baseline characteristics

	NCA (*n* = 3230)	DCA (*n* = 705)	PCI (*n* = 652)	Total (*n* = 4587)	*p*-value
**Age**					< 0.001
Median [Q1, Q3]	78 [68, 84]	76 [63, 82]	75 [64, 81]	77 [67, 83]	
**Gender**					< 0.001
Male	1854 (57.4%)	417 (59.1%)	473 (72.5%)	2744 (59.8%)	
Female	1376 (42.6%)	288 (40.9%)	179 (27.5%)	1843 (40.2%)	
**Troponin**,** ng/L**					< 0.001
Median [Q1, Q3]	31[20, 55]	32 [22, 61]	59 [28, 203]	33 [21, 63]	
N missing	3	0	0	3	
**Creatinine**,** mg/dl**					< 0.001
Median [Q1, Q3]	1.1 [0.9, 1.6]	1.0 [0.88, 1.3]	1.0 [0.88, 1.4]	1.1 [0.9, 1.5]	
N missing	3	1	0	4	
**Hospital Duration**,** days**					0.008
Median [Q1, Q3]	2 [0, 9]	5 [2, 11]	4 [2, 10]	3.0 [1, 10]	
**Discharges**					< 0.001
Regular discharge	2412 (74.7%)	640 (90.8%)	581 (89.1%)	3633 (79.2%)	
Discharged to external hospital	356 (11.0%)	39 (5.5%)	31 (4.8%)	426 (9.3%)	
Discharged against medical advice	99 (3.0%)	5 (0.7%)	3 (0.5%)	107 (2.3%)	
Discharged to rehabilitation	75 (2.3%)	2 (0.3%)	9 (1.4%)	86 (1.9%)	
Transferred to another hospital	11 (0.3%)	1 (0.1%)	1 (0.2%)	13 (0.3%)	
Transferred to psychiatry	10 (0.3%)	0 (0.0%)	0 (0.0%)	10 (0.2%)	
Transferred to collaborative work	1 (0.0%)	0 (0.0%)	0 (0.0%)	1 (0.0%)	
Transferred to weaning hospital	1 (0.0%)	0 (0.0%)	0 (0.0%)	1 (0.0%)	
Other discharge	9 (0.2%)	2 (0.3%)	0 (0.0%)	11 (0.2%)	
**Urgency by MTS**					0.196
Blue	24 (0.9%)	10 (1.6%)	9 (1.6%)	43 (1.1%)	
Yellow	1724 (61.8%)	377 (61.0%)	342 (59.9%)	2443 (61.4%)	
Green	556 (19.9%)	123 (19.9%)	108 (18.9%)	787 (19.8%)	
Orange	446 (16.0%)	105 (17.0%)	107 (18.7%)	658 (16.5%)	
Red	39 (1.4%)	3 (0.5%)	5 (0.9%)	47 (1.2%)	
N missing	441	87	81	609	
**In-hospital death**	256 (7.9%)	16 (2.3%)	27 (4.1%)	299 (6.5%)	< 0.001

Abbreviations: DCA, diagnostic coronary angiography; MTS, Manchester Triage System, NCA, no coronary angiography; PCI, percutaneous coronary intervention; ; Q, Quartile;

**Table 2:** Complaint pictures

Complaint picture	NCA (*n* = 3230)	DCA (*n* = 705)	PCI (*n* = 652)	Total (*n* = 4587)	*p*-value < 0.001
Malaise	1104 (39.6%)	210 (34.0%)	166 (29.1%)	1480 (37.2%)	
Breathing problem	647 (23.2%)	135 (21.8%)	101 (17.7%)	883 (22.2%)	
Chest pain	159 (5.7%)	113 (18.3%)	160 (28.0%)	432 (10.9%)	
Palpitations	141 (5.1%)	68 (11.0%)	53 (9.3%)	262 (6.6%)	
Collapse	112 (4.0%)	21 (3.4%)	20 (3.5%)	153 (3.8%)	
Abdominal pain	117 (4.2%)	14 (2.3%)	18 (3.2%)	149 (3.7%)	
General indicators	114 (4.1%)	8 (1.3%)	11 (1.9%)	133 (3.3%)	
Extremity problems	78 (2.8%)	13 (2.1%)	10 (1.8%)	101 (2.5%)	
Falls	60 (2.2%)	8 (1.3%)	13 (2.3%)	81 (2.0%)	
Gastrointestinal bleeding	52 (1.9%)	1 (0.2%)	2 (0.4%)	55 (1.4%)	
Seizure	47 (1.7%)	5 (0.8%)	1 (0.2%)	53 (1.3%)	
Diarrhea and vomiting	32 (1.1%)	1 (0.2%)	0 (0.0%)	33 (0.8%)	
Head injury	25 (0.9%)	3 (0.5%)	3 (0.5%)	31 (0.8%)	
Headache	23 (0.8%)	5 (0.8%)	2 (0.4%)	30 (0.8%)	
Overdose and poisoning	11 (0.4%)	1 (0.2%)	0 (0.0%)	12 (0.3%)	
Drunk appearance	6 (0.2%)	2 (0.3%)	2 (0.4%)	10 (0.3%)	
Conspicuous behavior	7 (0.3%)	1 (0.2%)	0 (0.0%)	8 (0.2%)	
Diabetes	6 (0.2%)	0 (0.0%)	2 (0.4%)	8 (0.2%)	
Back pain	9 (0.3%)	4 (0.6%)	2 (0.4%)	15 (0.4%)	
Asthma	5 (0.2%)	1 (0.2%)	0 (0.0%)	6 (0.2%)	
Psychiatric illness	6 (0.2%)	0 (0.0%)	1 (0.2%)	7 (0.2%)	
Eye problems	5 (0.2%)	0 (0.0%)	2 (0.4%)	7 (0.2%)	
Allergy	3 (0.1%)	2 (0.3%)	0 (0.0%)	5 (0.1%)	
Urological problems	5 (0.2%)	0 (0.0%)	0 (0.0%)	5 (0.1%)	
Wounds	4 (0.1%)	0 (0.0%)	0 (0.0%)	4 (0.1%)	
Facial problems	3 (0.1%)	0 (0.0%)	0 (0.0%)	3 (0.1%)	
Sore throat	1 (0.0%)	0 (0.0%)	1 (0.2%)	2 (0.1%)	
Abscesses and local infections	1 (0.0%)	1 (0.2%)	0 (0.0%)	2 (0.1%)	
Torso injury	2 (0.1%)	0 (0.0%)	0 (0.0%)	2 (0.1%)	
Resuscitation	0 (0.0%)	1 (0.2%)	0 (0.0%)	1 (0.0%)	
Severe trauma	0 (0.0%)	0 (0.0%)	1 (0.2%)	1 (0.0%)	
Dental problems	1 (0.0%)	0 (0.0%)	0 (0.0%)	1 (0.0%)	
Skin rashes	1 (0.0%)	0 (0.0%)	0 (0.0%)	1 (0.0%)	
N missing	441 (13.7%)	87 (12.3%)	81 (12.4%)	609 (13.3%)	

Abbreviations: DCA, diagnostic coronary angiography; NCA, no coronary angiography; PCI, percutaneous coronary intervention;

Approval for retrospective data analysis was granted by the Ethics Committee of the Technical University of Munich (2023-416-S-NP). The authors had no access to information that could identify individual participants during or after data collection. Data were fully anonymized before being accessed. This study was performed in line with the principles of the Declaration of Helsinki.

### Manchester Triage System (MTS)

The Manchester Triage System (MTS) is extensively used across Europe to assist nurses in prioritizing patients based on their presenting symptoms [5, 6]. Patients are assigned to one of five urgency categories: blue, green, yellow, orange or red, which determine the immediacy of physician intervention. This categorization is crucial for effective patient management in the ED. The distribution of patients across these categories is detailed in Table 1.

### Laboratory methods

Upon admission to the ED, blood samples were collected from all patients. Hs-cTnT levels were measured using an electrochemiluminescence immunoassay (ECLIA) on the Roche Cobas 8000 E801 chemistry analyzer (Roche Diagnostics, Mannheim, Germany). The Limit of Blank (LoB) of the “Elecsys Troponin T hs” assay which is used in clinical care at the Klinikum rechts der Isar, Munich, was 2.5 ng/L, the Limit of Detection (LoD) 3 ng/L and the Limit of Quantitation (LoQ) was 13 ng/L. Creatinine levels were assessed using an enzymatic method on the Roche Cobas 8000 C702 chemistry analyzer (Roche Diagnostics, Mannheim, Germany). For both hs-cTnT and creatinine, the initial values obtained at patient presentation were retrospectively analyzed.

### Statistical analysis

Quantitative data are described by median, 1st and 3rd quartile. For categorical data, absolute and relative frequencies are shown. Analysis of variance (ANOVA) was performed to compare distributions of quantitative data between patients who received NCA, DCA or PCI. For comparison of distributions of categorical data between these patient groups, χ^2^ tests were conducted. All statistical tests were performed two-sided and for all tests a significance level of α = 5% was used.

## Results

### Patient population

Applying the above-mentioned exclusion criteria shown in Fig. 1, we were able to include data from 4.587 patients in our retrospective descriptive analysis. Of these, 70.4% (3.230/4.587) received NCA, 15.4% (705/4.587) received DCA and 14.2% (652/4.587) received PCI. Table 1 shows the baseline characteristics of the comprised study population.

The median age was 77 years, with 40.2% being female. Hs-cTnT levels were significantly higher in patients who underwent PCI compared to those who received DCA or NCA (PCI: median 59 ng/L, 1st to 3rd quartile: [28; 202]; DCA: 32 ng/L [22; 61]; NCA: 31 ng/L [20; 55], *p* < 0.001). Creatinine levels were comparable between the groups (total median 1.1 mg/dl [0.9; 1,5]). Hospital duration was significantly longer for patients who underwent DCA compared to the other groups (DCA: 5 days [2.0; 11.0]; PCI: 4 days [1.0; 10.0], NCA: 2 days [0.0; 9.0], *p* < 0.008). There were no significant differences between the groups regarding MTS categorization (*p* = 0.196). However, patients with NCA (1.4%, 39/3.230) were more frequently categorized as “red” compared to the DCA (0.5%, 3/705) and the PCI group (0.9%, 5/652). Interestingly, in-hospital death was 6.5% (299/4587) overall, with a higher mortality rate observed in the NCA group (7.9%, 256/3230) compared to the DCA (2.3%, 16/705) and PCI group (4.1%, 27/652; *p* < 0.001). There was no difference regarding in-hospital mortality between male and female patients among the groups (see Table S1).

Table 2 shows the presenting complaints of all analysed patients. Overall, 10.9% (432/4.587) patients presented with chest pain. However, the prevalence of chest pain was more frequent in patients who underwent PCI (28.0%, 160/652) compared to the DCA group (18.3%, 113/705) and NCA group (5.7%, 159/3230), (*p* < 0.001). Palpitations were described more frequently in patients who underwent coronary angiography (DCA: 11.0%, 68/705; PCI: 9.3%, 53/652) compared to patients who did not undergo coronary angiography (NCA: 5.1%, 141/3230). However, breathing problems occurred more frequently in the NCA group (23.2%, 647/3230) compared to the PCI group (17.7%, 101/652) and DCA group (21.8%, 135/705), (*p* < 0.001). Also, collapse was more frequent in patients in the NCA groups (4.0%, 112/3230) compared to the DCA group (3.4%, 21/705), PCI group (3.5%, 20/652), (*p* < 0.001).

### Incidence and localization of myocardial infarction

**Fig. 2:**
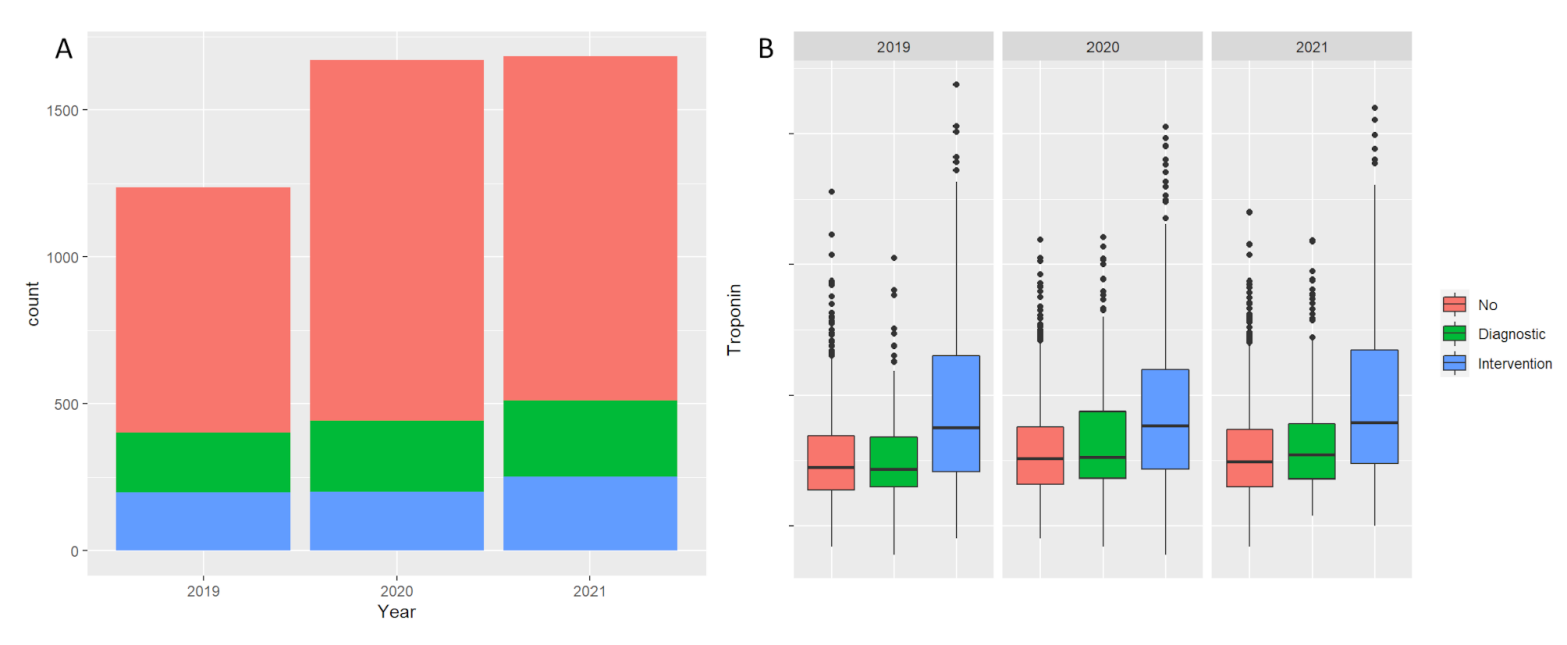
**A**: Amount of emergency department patients with elevated hs-cTnT between 2019 and 2021. **B**: Development of hs-cTnT values between 2019 and 2021

Emergency department patients with elevated hs-cTnT levels exhibited a consistent distribution to the different groups from 2019 to 2021 (see Table 3). Numerically, the count of patients with elevated hs-cTnT increased over the years, as also illustrated in Fig. 2A.

**Table 3:** Count of patients with elevated hs-cTnT between 2019 and 2021

	2019 (*n* = 1236)	2020 (*n* = 1669)	2021 (*n* = 1682)	Total (*n* = 4587)
**NCA**	832 (67.3%)	1227 (73.5%)	1171 (69.6%)	3230 (70.4%)
**DCA**	205 (16.6%)	242 (14.5%)	258 (15.3%)	705 (15.4%)
**PCI**	199 (16.1%)	200 (12.0%)	253 (15.0%)	652 (14.2%)

Abbreviations: DCA, diagnostic coronary angiography; NCA, no coronary angiography; PCI, percutaneous coronary intervention;

The distribution of hs-cTnT values of the analysed groups are graphically depicted in Fig. 2B. Patients who underwent PCI consistently showed higher hs-cTnT levels over the study period compared to those in other treatment groups (PCI: median 59 ng/L, 1st to 3rd quartile: [28; 202]; DCA: 32 ng/L [22; 61]; NCA: 31 ng/L [20; 55], *p* < 0.001, see also Table 1).

The combined incidence of ST-elevation myocardial infarction (ICD-10 codes I21.0, I21.1, I21.2) and non-ST-elevation myocardial infarction (ICD-10 codes I21.4, I21.9) was 12.1% in 2019, 10.5% in 2020, and 13.2% in 2021; however, it did not differ between the years (*p* = 0.176, see Table 4).

**Table 4:** Localization of myocardial infarction

	2019 (*n* = 1236)	2020 (*n* = 1669)	2021 (*n* = 1682)	Total (*n* = 4587)	*p*-value 0.176
**I21.0**	10 (0.8%)	14 (0.8%)	16 (1.0%)	40 (0.9%)	
**I21.1**	14 (1.1%)	10 (0.6%)	14 (0.8%)	38 (0.8%)	
**I21.2**	8 (0.6%)	3 (0.2%)	5 (0.3%)	16 (0.3%)	
**I21.4**	118 (9.5%)	147 (8.8%)	187 (11.1%)	452 (9.9%)	
**I21.9**	1 (0.1%)	1 (0.1%)	0 (0.0%)	2 (0.0%)	
**Other#**	1085 (87.8%)	1494 (89.5%)	1460 (86.8%)	4039 (88.1%)	

Abbreviations: I21.0 Acute myocardial infarction of anterior wall; I21.1 Acute myocardial infarction of inferior wall; I21.2, Acute myocardial infarction of other sites, I21.4 Acute subendocardial myocardial infarction I21.9, Acute myocardial infarction, unspecified. **#** other than myocardial infarction;

### In-hospital mortality

In-hospital mortality increased with higher levels of hs-cTnT, as detailed in Table S2. Additionally, among patients with elevated hs-cTnT, those with COVID-19 experienced higher in-hospital mortality (77/290, 26.6%) compared to those without COVID-19 (222/4297, 5.2%). Of the 77 COVID-19 positive patients who died in-hospital, the majority (75/77, 97.4%) were in the NCA group, with one patient each in the DCA group (1/77, 1.3%) and PCI group (1/77, 1.3%). Notably, in-hospital mortality in the NCA group was significantly higher for patients with COVID-19 compared to those without COVID-19 (75/282, 26.6% vs. 181/2948, 6.1%; *p* < 0.001). Furthermore, we observed that in-hospital mortality was significantly elevated among unstable patients, defined by MTS categorization to orange or red, with 10.8% compared to 5.5% in stable patients, categorized as blue, green or yellow (*p* < 0.001, see Table S2). There was no significant difference in in-hospital mortality between male and female patients across the different groups, as illustrated in Table S1.

## Discussion

This retrospective descriptive study was conducted in a high-volume interdisciplinary emergency department in Munich, Germany, between 2019 and 2021. To our knowledge, it is the first study to characterize such a large cohort of emergency patients with elevated hs-cTnT and assess their subsequent clinical outcomes. Among the 4.587 analysed patients with elevated hs-cTnT levels, 70.4% did not undergo coronary angiography (NCA), 15.4% underwent diagnostic coronary angiography (DCA), and 14.2% underwent percutaneous coronary intervention (PCI). Interestingly, only a small number of these patients presented with chest pain symptoms. Yet, breathing problems and collapse occurred more frequently in patients who received no coronary angiography in the further clinical course. Notably, the in-hospital mortality rate was higher among these patients who did not undergo coronary angiography.

Overall, a higher percentage of patients with elevated hs-cTnT received no coronary angiography (NCA) compared to those who underwent diagnostic coronary angiography (DCA) or percutaneous coronary intervention (PCI). In 2020, the year of the COVID-19 pandemic, almost 74% of patients with elevated hs-cTnT were treated conservatively. This rather conservative approach might be owed to the COVID-19 pandemic which significantly impacted our global healthcare systems [7]: According to a study by Tschaikowsky et al. [8], patient visits to the emergency department in Munich decreased during the pandemic, likely due to patients’ fear of contracting COVID-19 in hospitals. Concurrently, invasive procedures and surgeries were performed more restrictively [9]. Interestingly, the count of patients with elevated hs-cTnT levels increased between 2019 and 2021 in our study. The rising number of COVID-19 cases during this period could have contributed to the increase in hs-cTnT levels: potential mechanisms include cardiovascular injury, inflammatory myocarditis, stress-induced cardiomyopathy, microvascular dysfunction, and thrombosis due to hypercoagulability or systemic inflammation destabilizing coronary artery plaques [10–13]. In our study we found that among patients with elevated hs-cTnT, those with COVID-19 experienced higher in-hospital mortality compared to those without COVID-19. Interestingly, the majority of these COVID-19 positive patients who died in-hospital received no invasive coronary angiography. The in-hospital mortality in the NCA group was significantly higher for patients with COVID-19 compared to those without COVID-19. However, it remains unclear whether the increased mortality of these patients was attributable to the severity of the COVID-19 infection or the absence of further invasive angiography.

Further, we observed that overall only 10.9% of all analysed patients presented with chest pain, while the overall in-hospital mortality rate was high at 6.5%. Although the prevalence of chest pain, the most common symptom for AMI, was more frequent in patients who underwent PCI, in-hospital mortality was higher in patients who did not receive coronary angiography. Notabaly, in the latter, breathing problems and collapse occurred more frequently compared to the other groups. Against this background, the CHARITEM study reported similar findings, with an overall in-hospital mortality rate of 4.7% among all in-patients [14]. This study also found that patients presenting with chest pain had significantly lower in-hospital mortality compared to those with dyspnea or even abdominal pain as their primary symptom. The authors attributed this to the well-established, highly standardized protocols for managing chest pain, which include greater awareness at admission and specialized care in chest pain units, leading to better patient outcomes [14–17]. Our findings highlight the heterogeneity of symptoms among emergency patients with elevated hs-cTnT.

In this context, risk assessment tools for incoming emergency patients have been developed to ensure more effective patient management. The Manchester Triage System [5] is performed upon admission and uses a software application to categorize patients into one of five urgency levels. We observed that patients who did not undergo coronary angiography were more frequently assigned to the “red” category, indicating the highest level of urgency. Further, in-hospital mortality was higher in unstable patients compared to stable patients. Delays in initiating appropriate therapy and losing valuable time may consequently increase the risk of mortality. Life-threatening conditions can elevate troponin levels independently of direct coronary artery involvement and are also associated with high mortality rates [18]. Indeed, we found that higher hs-cTnT levels were associated with higher in-hospital mortality rates. However, it is crucial to recognize that elevated hs-cTnT levels can occur in various conditions due to reduced oxygen supply to the myocardium [19]. These conditions may include sepsis, systemic inflammatory response syndrome, end-stage renal disease, pulmonary embolism, hypotension, hypovolemia or tachyarrhythmias [19–23].

## Conclusion

The mortality rate among emergency patients with elevated hs-cTnT who did not undergo coronary angiography was notably high. There is a widespread use of hs-cTnT in emergency departments, not only for suspected cardiac conditions.

### Limitations

This study is subject to limitations inherent in retrospective descriptive data analysis. A broader spectrum of patient characteristics would be desirable; specifically, the inclusion of electrocardiograms at admission and a deeper understanding of the underlying conditions (e.g., pneumonia, sepsis, cancer) associated with higher mortality in the NCA group. Unfortunately, these details could not be extracted from our current information system. Nevertheless, this study represents the largest to date analyzing emergency department patients with elevated hs-cTnT over a three-year period.

## Electronic supplementary material

Below is the link to the electronic supplementary material.

Supplementary Material 1

## Data Availability

No datasets were generated or analysed during the current study.

## Abbreviations

AMI: Acute myocardial infarction
DCA: Diagnostic coronary angiography
ED: Emergency department
Hs-cTnT: High-sensitive cardiac troponin T
MTS: Manchester Triage System
NCA: No coronary angiography
PCI: Percutaneous coronary intervention

## References

[1] Savioli G, Ceresa IF, Gri N, Bavestrello Piccini G, Longhitano Y, Zanza C et al. Emergency Department Overcrowding: Understanding the Factors to Find Corresponding Solutions. J Pers Med. 2022;12(2). Epub 2022/02/26. 10.3390/jpm12020279. PubMed PMID: 35207769; PubMed Central PMCID: PMCPMC8877301. 10.3390/jpm12020279 PMC8877301 35207769

[2] Mechanic OJ, Gavin M, Grossman SA. Acute myocardial infarction. StatPearls. Treasure Island (FL): StatPearls Publishing Copyright © 2023. StatPearls Publishing LLC.; 2023.

[3] Ko DT, Dattani ND, Austin PC, Schull MJ, Ross JS, Wijeysundera HC, et al. Emergency Department Volume and outcomes for patients after chest Pain Assessment. Circ Cardiovasc Qual Outcomes. 2018;11(11):e004683. 10.1161/circoutcomes.118.004683. Epub 2018/10/26. 30354285 10.1161/CIRCOUTCOMES.118.004683

[4] Sandoval Y, Apple FS, Mahler SA, Body R, Collinson PO, Jaffe AS, AHA/ACC/ASE/CHEST. /SAEM/SCCT/SCMR guidelines for the evaluation and diagnosis of acute chest Pain. Circulation. 2022;146(7):569–81. 10.1161/circulationaha.122.059678. Epub 2022/07/02. 35775423 10.1161/CIRCULATIONAHA.122.059678

[5] Gräff I, Goldschmidt B, Glien P, Bogdanow M, Fimmers R, Hoeft A, et al. The German version of the Manchester Triage System and its quality criteria–first assessment of validity and reliability. PLoS ONE. 2014;9(2):e88995. 10.1371/journal.pone.0088995. Epub 2014/03/04. 24586477 10.1371/journal.pone.0088995 PMC3933424

[6] Kevin Mackway-Jones JM, Jill W. Emergency triage: Manchester Triage Group. Ltd: Wiley; 2014. 10.1002/9781118299029.

[7] Filip R, Gheorghita Puscaselu R, Anchidin-Norocel L, Dimian M, Savage WK. Global Challenges To Public Health Care Systems during the COVID-19 pandemic: a review of pandemic measures and problems. J Pers Med. 2022;12(8). 10.3390/jpm12081295. PubMed PMID: 36013244; PubMed Central PMCID: PMCPMC9409667. Epub 2022/08/27. 10.3390/jpm12081295 PMC9409667 36013244

[8] Tschaikowsky T, von Becker A, Consalvo S, Pflüger P, Barthel P, Spinner CD, et al. [Numbers of emergency room patients during the COVID-19 pandemic]. Notf Rett Med. 2021;24(6):943–52. 10.1007/s10049-020-00757-w. Epub 2020/08/25. 32837303 10.1007/s10049-020-00757-w PMC7341467

[9] Mattingly AS, Rose L, Eddington HS, Trickey AW, Cullen MR, Morris AM, et al. Trends in US Surgical procedures and Health Care System response to policies curtailing Elective Surgical Operations during the COVID-19 pandemic. JAMA Netw Open. 2021;4(12):e2138038. 10.1001/jamanetworkopen.2021.38038. Epub 2021/12/09. 34878546 10.1001/jamanetworkopen.2021.38038 PMC8655602

[10] Libby P, Loscalzo J, Ridker PM, Farkouh ME, Hsue PY, Fuster V, et al. Inflammation, immunity, and infection in Atherothrombosis: JACC Review topic of the Week. J Am Coll Cardiol. 2018;72(17):2071–81. PubMed PMID: 30336831; PubMed Central PMCID: PMCPMC6196735. 30336831 10.1016/j.jacc.2018.08.1043 PMC6196735

[11] Modin D, Claggett B, Sindet-Pedersen C, Lassen MCH, Skaarup KG, Jensen JUS, et al. Acute COVID-19 and the incidence of ischemic stroke and Acute Myocardial Infarction. Circulation. 2020;142(21):2080–2. 10.1161/circulationaha.120.050809. Epub 2020/10/16. 33054349 10.1161/CIRCULATIONAHA.120.050809 PMC7682795

[12] Bangalore S, Sharma A, Slotwiner A, Yatskar L, Harari R, Shah B, et al. ST-Segment Elevation in patients with Covid-19 - a Case Series. N Engl J Med. 2020;382(25):2478–80. 10.1056/NEJMc2009020. Epub 2020/04/18. 32302081 10.1056/NEJMc2009020 PMC7182015

[13] Bilaloglu S, Aphinyanaphongs Y, Jones S, Iturrate E, Hochman J, Berger JS, Thrombosis in Hospitalized Patients With COVID-19 in a New York City Health System. Jama. 2020;324(8):799–801. Epub 2020/07/24. doi: 10.1001/jama.2020.13372. PubMed PMID: 32702090; PubMed Central PMCID: PMCPMC7372509 distribution related to the ISCHEMIA Trial and in-kind donations for participating sites from AstraZeneca Pharmaceuticals and Arbor Pharmaceuticals; in-kind donations for participating sites from Abbott Vascular, Medtronic Inc, St Jude Medical Inc, Volcano Corp, Merck Sharp & Dohme Corp, Omron Healthcare Inc, and Amgen Inc; and grants from the National Heart, Lung, and Blood Institute for serving as chair of the ISCHEMIA study. Dr Berger reported receiving grants from AstraZeneca, personal fees from Janssen, and personal fees from Amgen outside the submitted work. No other disclosures were reported. 10.1001/jama.2020.13372 PMC7372509 32702090

[14] Mockel M, Searle J, Muller R, Slagman A, Storchmann H, Oestereich P, et al. Chief complaints in medical emergencies: do they relate to underlying disease and outcome? The Charité Emergency Medicine Study (CHARITEM). Eur J Emerg Med. 2013;20(2):103–8. 10.1097/MEJ.0b013e328351e609. Epub 2012/03/06. 22387754 10.1097/MEJ.0b013e328351e609

[15] Bhuiya FA, Pitts SR, McCaig LF. Emergency department visits for chest pain and abdominal pain: United States, 1999–2008. NCHS Data Brief. 2010;43:1–8. Epub 2010/09/22. PubMed PMID: 20854746. 20854746

[16] Keller T, Post F, Tzikas S, Schneider A, Arnolds S, Scheiba O, et al. Improved outcome in acute coronary syndrome by establishing a chest pain unit. Clin Res Cardiol. 2010;99(3):149–55. 10.1007/s00392-009-0099-9. Epub 2009/12/25. 20033695 10.1007/s00392-009-0099-9

[17] Furtado MV, Cardoso A, Patrício MC, Rossini AP, Campani RB, Meotti C, et al. Influence of implementation of a chest pain unit on acute coronary syndrome outcomes. J Emerg Med. 2011;40(5):557–64. PubMed PMID: 20022199. 20022199 10.1016/j.jemermed.2009.08.062

[18] Matsunaga N, Yoshioka Y, Fukuta Y. Extremely high troponin levels induced by septic shock: a case report. J Med Case Rep. 2021;15(1):466. 10.1186/s13256-021-03027-6. Epub 2021/09/12. 34507615 10.1186/s13256-021-03027-6 PMC8433049

[19] Akwe JHB, Kim E, Miller A. A review of Cardiac and Non-cardiac causes of Troponin Elevation and Clinical Relevance Part II non Cardiac causes. J Cardiol Curr Res. 2018.

[20] Ammann P, Maggiorini M, Bertel O, Haenseler E, Joller-Jemelka HI, Oechslin E, et al. Troponin as a risk factor for mortality in critically ill patients without acute coronary syndromes. J Am Coll Cardiol. 2003;41(11):2004–9. 10.1016/s0735-1097(03)00421-2. Epub 2003/06/12. 12798573 10.1016/s0735-1097(03)00421-2

[21] Bakshi TK, Choo MK, Edwards CC, Scott AG, Hart HH, Armstrong GP. Causes of elevated troponin I with a normal coronary angiogram. Intern Med J. 2002;32(11):520-5. Epub 2002/11/05. 10.1046/j.1445-5994.2002.00270.x. PubMed PMID: 12412934. 10.1046/j.1445-5994.2002.00270.x 12412934

[22] Wright RS, Williams BA, Cramner H, Gallahue F, Willmore T, Lewis L, et al. Elevations of cardiac troponin I are associated with increased short-term mortality in noncardiac critically ill emergency department patients. Am J Cardiol. 2002;90(6):634–6. 10.1016/s0002-9149(02)02570-5. Epub 2002/09/17. 12231092 10.1016/s0002-9149(02)02570-5

[23] Zellweger MJ, Schaer BA, Cron TA, Pfisterer ME, Osswald S. Elevated troponin levels in absence of coronary artery disease after supraventricular tachycardia. Swiss Med Wkly. 2003;133(31–32):439–41. 10.4414/smw.2003.10288. Epub 2003/10/17. PubMed PMID: 14562187. 14562187 10.4414/smw.2003.10288

