# Supplementary material for: Elevated high-sensitive cardiac troponin T in emergency department patients: insights from a retrospective descriptive cohort study
Source: Int J Emerg Med. 2024 Oct 7;17:141. doi: 10.1186/s12245-024-00735-w (PMC11457446; doi:10.1186/s12245-024-00735-w)
Supplement: Supplementary file 1 — Supplementary Material 1 [file 12245_2024_735_MOESM1_ESM.docx]

**Table S1: Baseline characteristics stratified by gender**

|  |  | **Male**  **(n=2744)** | **Female**  **(n=1843)** | **Total**  **(n=4587)** | **p-value** |
| --- | --- | --- | --- | --- | --- |
| **NCA** | **Age** |  |  |  | <0.001 |
|  | Median [Q1, Q3] | 75 [65, 82] | 80 [72, 86] | [68, 84] |  |
|  | **Troponin, ng/L** |  |  |  | 0.513 |
|  | Median [Q1, Q3] | 32 [20, 57] | 30 [20, 51] | 31 [20, 55] |  |
|  | **Creatinine, mg/dl** |  |  |  | <0.001 |
|  | Median [Q1, Q3] | 1.2 [0.9, 1.7] | 1.0 [0.8, 1.5] | 1.1 [0.9, 1.6] |  |
|  | **Duration hospital, days** |  |  |  | 0.461 |
|  | Median [Q1, Q3] | 2 [1, 9] | 3 [0, 10] | 2 [0, 9] |  |
|  | **Urgency by MTS** |  |  |  | 0.343 |
|  | Blue | 12 (0.7%) | 12 (1.0%) | 24 (0.9%) |  |
|  | Green | 329 (20.5) | 227 (19.2%) | 556 (19.9%) |  |
|  | Yellow | 972 (60.5%) | 752 (63.3%) | 1724 (61.8%) |  |
|  | Orange | 272 (16.9) | 174 (14.7%) | 446 (16.0%) |  |
|  | Red | 21 (1.3%) | 18 (1.5%) | 39 (1.4%) |  |
|  | N missing | 248 | 193 | 441 |  |
|  | **In-hospital death** | 155 (8.4%) | 101 (7.3%) | 256 (7.9%) | 0.289 |
| **DCA** | **Age** |  |  |  | <0.001 |
|  | Median [Q1, Q3] | 73 [60, 80] | 78 [70.75, 83] | 76 [63, 82] |  |
|  | **Troponin, ng/L** |  |  |  | 0.259 |
|  | Median [Q1, Q3] | 32 [22, 58] | 32 [21, 73] | 32 [22, 60] |  |
|  | **Creatinine, mg/dl** |  |  |  | <0.001 |
|  | Median [Q1, Q3] | 1.1 [0.9, 1.4] | 0.9 [0.7, 1.2] | 1.0 [0.875, 1.3] |  |
|  | **Duration hospital, days** |  |  |  | 0.266 |
|  | Median [Q1, Q3] | 5 [2, 12] | 5 [2, 11] | 5 [2, 11] |  |
|  | **Urgency by MTS** |  |  |  | 0.625 |
|  | Blue | 5 (1.4%) | 5 (2.0%) | 10 (1.6%) |  |
|  | Green | 75 (20.3%) | 48 (19.3%) | 123 (19.9%) |  |
|  | Yellow | 225 (61.0%) | 152 (61.0%) | 377 (61.0%) |  |
|  | Orange | 61 (16.5%) | 44 (17.7%) | 105 (17.0%) |  |
|  | Red | 3 (0.8%) | 0 (0.0%) | 3 (0.5%) |  |
|  | N missing | 48 | 39 | 87 |  |
|  | **In-hospital death** | 8 (1.9%) | 8 (2.8%) | 16 (2.3%) | 0.451 |
| **PCI** | **Age** |  |  |  | <0.001 |
|  | Median [Q1, Q3] | 73 [61, 80] | 79 [73, 83] | 75 [64, 81] |  |
|  | **Troponin, ng/L** |  |  |  | 0.800 |
|  | Median [Q1, Q3] | 60 [29, 213] | 56 [26, 177] | 59 [28, 203] |  |
|  | **Creatinine, mg/dl** |  |  |  | 0.457 |
|  | Median [Q1, Q3] | 1.1 [0.9, 1.4] | 0.9 [0.7, 1.3] | 1.0 [0.875, 1.4] |  |
|  | **Duration hospital, days** |  |  |  | 0.586 |
|  | Median [Q1, Q3] | 4 [1, 9] | 3 [2, 12.5] | 4 [1.75, 10] |  |
|  | **Urgency by MTS** |  |  |  | 0.477 |
|  | Blue | 8 (2.0%) | 1 (0.6%) | 9 (1.6%) |  |
|  | Green | 76 (18.7%) | 32 (19.5%) | 108 (18.9%) |  |
|  | Yellow | 243 (59.7%) | 99 (60.4%) | 342 (59.9%) |  |
|  | Orange | 75 (18.4%) | 32 (19.5%) | 107 (18.7%) |  |
|  | Red | 5 (1.2%) | 0 (0.0%) | 5 (0.9%) |  |
|  | N missing | 66 | 15 | 81 |  |
|  | **In-hospital death** | 19 (4.0%) | 8 (4.5%) | 27 (4.1%) | 0.796 |

**Abbreviations:** DCA, diagnostic coronary angiography; MTS, Manchester Triage System, NCA, no coronary angiography; PCI, percutaneous coronary intervention; Q, Quartile;

**Table S2: In-hospital mortality**

|  | **In-hospital mortality**  **(n=299)** | **p-value** |
| --- | --- | --- |
| **Urgency by MTS** |  | <0.001 |
| Blue | 3/43 (7.0%) |  |
| Green | 34/787 (4.3%) |  |
| Yellow | 142/2443 (5.8%) |  |
| Orange | 64/658 (9.7%) |  |
| Red | 12/47 (25.5%) |  |
| N missing | 44 |  |
| **Stable vs. unstabile patients** |  | <0.001 |
| Blue/yellow/green | 179/3273 (5.5%) |  |
| Orange/red | 76/705 (10.8%) |  |
| N missing | 44 |  |
| **Hs-cTnT, ng/L** |  | <0.001 |
| >14 - <30 | 265/4348 (6.1%) |  |
| 30 - <50 | 11/88 (12.5%) |  |
| ≥ 50 | 23/148 (15.5%) |  |

**Abbreviations:** Hs-cTnT, high-sensitive cardiac troponin T; MTS, Manchester Triage System;
